# Malaria vector control tools in emergency settings: What do experts think? Results from a DELPHI survey

**DOI:** 10.1186/s13031-021-00424-y

**Published:** 2021-12-20

**Authors:** Christophe Boëte, Sakib Burza, Estrella Lasry, Silvia Moriana, William Robertson

**Affiliations:** 1 ISEM, CNRS, IRD, EPHE Place Eugene Bataillon CC65, Univ Montpellier, 34095 Montpellier, France; 2 Médecins Sans Frontières Spain, Carrer de Zamora, 54, 08005 Barcelona, Spain; 3 Médecins Sans Frontières, New Delhi, India; 4 London School of Hygiene and Tropical Medicine, London, UK

**Keywords:** DELPHI survey, Emergency settings, Malaria, Vector control, Innovation, Public health, Perception

## Abstract

**Background:**

The use and implementation of novel tools for malaria control such as long lasting impregnated bednets (LLINs) and Indoor Residual Spraying (IRS) over the last decade has contributed to a substantial reduction in malaria burden globally. However numerous challenges exist particularly in relation to vector control in emergency settings. This study seeks to explore expert opinion on the utility of existing tools within the emergency context setting and to better understand the attitude towards emerging and innovative tools (including Genetically Modified Mosquitoes) to augment current approaches.

**Methods:**

80 experts in the field of malaria and vector control were invited to participate in a two-round Delphi survey. They were selected through a combination of literature (academic and policy publications) review and snowball sampling reflecting a range of relevant backgrounds including vector control experts, malaria programme managers and emergency response specialists. The survey was conducted online through a questionnaire including the possibility for free text entry, and concentrated on the following topics:

1. Utility and sustainability of current vector control tools, both in and outside emergency settings
2. Feasibility, utility and challenges of emerging vector control tools, both in and outside emergency settings
3. Current and unmet research priorities in malaria vector control and in malaria control in general.

**Results:**

37 experts completed the first round and 31 completed the second round of the survey. There was a stronger consensus about the increased utility of LLIN compared to IRS in all settings, while insecticide-treated covers and blankets ranked very high only in emergency settings. When considering the combination of tools, the ones deemed most interesting always involved LLINs and IRS regardless of the setting, and the acceptability and the efficacy at reducing transmission are essential characteristics. Regarding perceptions of tools currently under development, consensus was towards improvement of existing tools rather than investing in novel approaches and the majority of respondents expressed distrust for genetic approaches.

**Conclusion:**

Malaria vector control experts expressed more confidence for tools whose efficacy is backed up by epidemiological evidence, hence a preference for the improvement rather than the combination of existing tools. Moreover, while several novel tools are under development, the majority of innovative approaches did not receive support, particularly in emergency settings. Stakeholders involved in the development of novel tools should involve earlier and raise awareness of the potential effectiveness amongst a wider range of experts within the malaria community to increase acceptability and improve early adoption once the evidence base is established.

**Supplementary Information:**

The online version contains supplementary material available at 10.1186/s13031-021-00424-y.

## Introduction

Malaria control relies heavily on the control of mosquito vectors, using mainly long lasting impregnated bednets (LLINs) and to a lesser extent Indoor Residual Spraying (IRS). The deployment of any method to prevent, test or treat malaria is subject to local human and environmental conditions. More than 90% of worldwide malaria-associated deaths currently occur in sub-Saharan Africa where the prevalence of complex chronic humanitarian emergency settings remains higher than anywhere else in the world [1, 2]. Effective malaria control must be able to be conducted in unstable and difficult conditions if short to mid-term goals of mortality reduction are to be met. However acute or protracted emergency conditions often preclude this to be done optimally and there remains a gap in the literature, particularly regarding the most efficient malaria-control approaches to deploy in a given context [3]. Such conditions require an emphasis on different or adapted tools with specific characteristics that may not be as relevant in more stable contexts, where access to the population is usually better, and the type of household allows for the use of existing tools developed mainly for stable settings.

In order to better understand the needs and challenges of malaria control in and outside humanitarian emergency contexts, a 2-stage Delphi survey of malaria control experts was conducted. The survey focused on humanitarian emergency contexts and vector control, and explored perceptions of utility and sustainability of existing and emerging strategies.

Delphi surveys are based on the principle that structured expert group participation from differing perspectives is more valid than individual judgements. They are conducted with a panel of experts through an iterative multi-stage process and can provide insight when observational or experimental data are limited [4, 5]. Typically, a Delphi survey involves a specified number of question rounds, each of which is followed by feedback on the degree of group consensus to participants. In theory, consecutive rounds are conducted to the stage where a group consensus (or not) is achieved.

## Methods

Participants in this study were subject experts with experience in the field of malaria control and/or emergency settings, and were selected through a literature review using the search terms ‘malaria’; ‘humanitarian’; ‘emergencies’ over the last 10 years. Grey literature was also screened from agencies implementing components of malaria control in humanitarian settings. Further participants were identified though snowballing sampling, i.e. the inclusion of experts following the recommendation by other participants for their expertise on the topic.

An email invitation to participate in the survey (Additional file 1: Mat 01) was sent in June 2018 to 80 experts, amongst whom 41 agreed to participate.

With the first-round questionnaire, (Additional file 2: Mat 02) the objective was to consider several major topics around malaria control while being as exhaustive as possible regarding the tools and techniques currently in use or under development: the utility and sustainability of current vector control tools, both in and outside emergency settings; the feasibility, utility and challenges of emerging vector control tools, both in and outside emergency settings; the current and unmet research priorities in malaria vector control and in malaria control in general.

The survey was sent in July 2018 through the online survey software QuestionPro (http://www.questionpro.com/) to the 41 experts willing to get involved. Several reminders were sent to non-responders before the first round of the survey was closed, by which 37 complete responses had been received (response rate of 46.2%). After consolidating responses, a second questionnaire was conducted in order to gather a consensus (if possible) between the remaining divergent opinions from the first round. Thus, this second survey round (Additional file 3: Mat 03) was sent to the 37 respondents, which in turn was completed by 31 experts, leading to an overall response rate of 38.7%.

The study was performed in compliance with the Helsinki Declaration. All participants were informed about the aim of this questionnaire and were free not to participate or to withdraw at any stage of the process. The replies were analysed in an anonymous manner.

## Results

### Type of research and experience

**Fig. 1:**
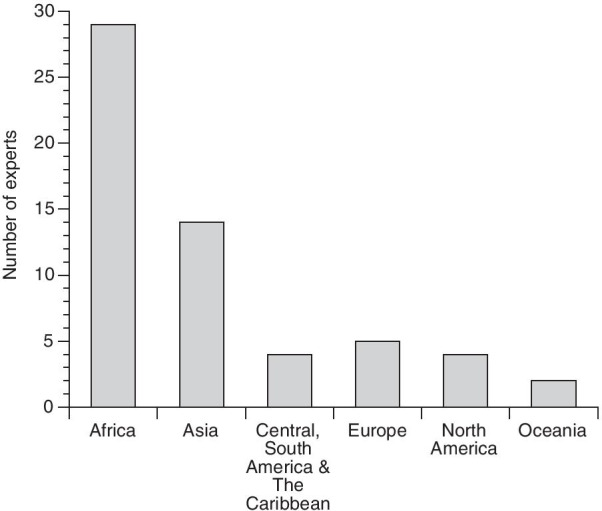
Number of participants according to the location where they spent most of their working time. Note that the total number exceeds the number of responders because it was possible to select multiple locations

All responders identified themselves as having experience in malaria vector control while 21 identified as additionally being experienced with emergency settings (ES). Regarding the working region, Africa (29/31) and Asia (14/31) were the most heavily represented (Fig. 1).

**Fig. 2:**
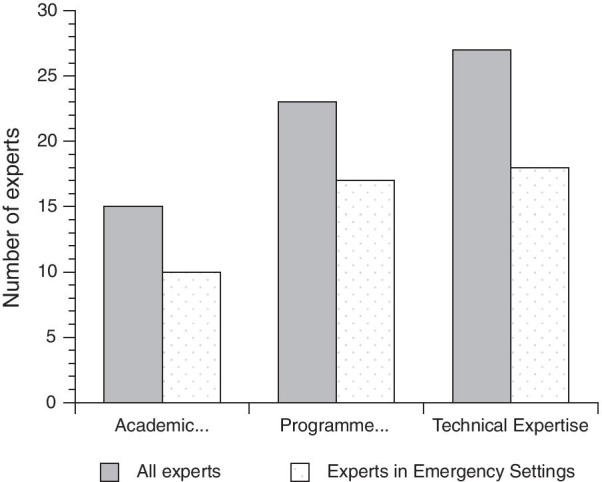
Type of expertise of the responders involved in the survey

Concerning participants self-reported areas of expertise, technical (27/31) and programmatic (23/31) expertise in malaria control were the most represented, while academic expertise was present in under half of the responders (15/31). The proportions (10/31, 17/31, 18/31) of participants reporting expertise in emergency settings was similar across the three domains (Fig. 2).

### Usefulness of current control tools

#### Usefulness of a single vector control tool

**Fig. 3:**
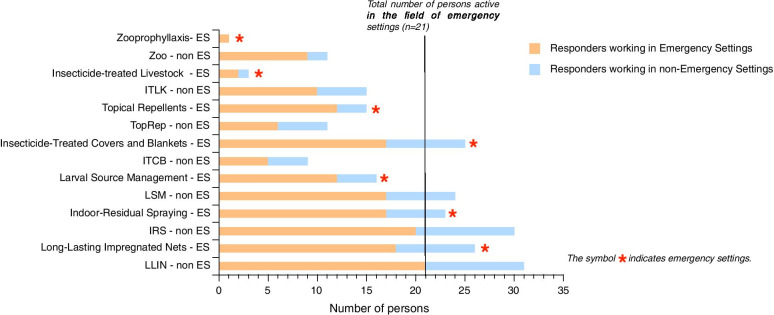
Interest of the current control tools in emergency and non-emergency settings for the experts involved in the survey. Responses are presented for all the experts (37) as well as for the ones (21) that have been declaring being experienced in emergency settings. *LLIN* Long-Lasting Impregnated Nets; *IRS*: Indoor-Residual Spraying; *LSM*/*LT* Larval Source Management/Larvicide Treatment; *ITCB* Insecticide-Treated Covers and Blankets; *TopRep* Topical Repellents; *ITLK* Insecticide-Treated LivestocK; *Zoo* Zooprophyllaxis

Participants were presented with a list of tools currently used for malaria vector control and were requested to value their relative utility in different settings, taking into account cost, ease of implementation and efficacy at reducing transmission (Fig. 3).

In emergency settings there was a clear consensus on the utility of LLIN (*Long-Lasting Impregnated Nets*) with more than 80% of participants considering them useful; this was even stronger in non-emergency settings. The support appeared slightly more situation-specific for IRS (Indoor-Residual Spraying) that is highly regarded in non-emergency settings (over 90%) with lower value (around 70%) in emergency settings. Regarding LSM/LT (Larval Source Management/ Larvicide Treatment) and Topical Repellents, they were considered of interest by about half of respondents, with no consensus on utility taking into account cost, ease of implementation and efficacy at reducing transmission. Regarding Insecticide-Treated Covers and Blankets (ITCB), while it was not considered of much interest in non-emergency settings (less than 30%), nearly 80% of responders considered it useful in emergency settings. This comes as no surprise as they are dedicated to people on the move and because of their demonstrated advantages in terms of protection and ease of deployment as shown in Afghan refugee camps [6] as well as in Kenya [7]. However, due to the higher cost of the item, some experts mentioned that more data on the efficacy of ITCB was needed before considering their deployment at scale.

**Fig. 4:**
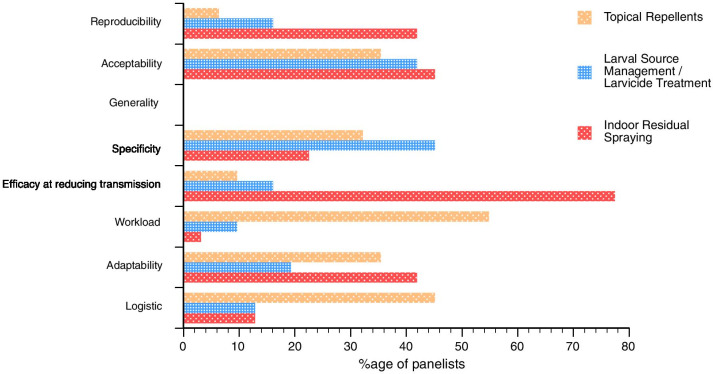
Characteristics of IRS (*IRS* Indoor-Residual Spraying); *LSM/LT* (Larval Source Management/Larvicide Treatment) and Topical Repellents in emergency settings

Explanations for the differences between contexts for IRS and the absence of a consensus for LSM/LT and Topical Repellents were explored further in the second round of the Delphi survey, in an attempt to determine the advantages and disadvantages of each considered tool in emergency settings (Fig. 4). For IRS, while there is a clear consensus amongst experts about its efficacy at reducing transmission, its effective deployment in emergency settings faces barriers such as difficult implementation logistics and high human resources workload requirements. Regarding LSM/LT and Topical Repellents, the agreement on their major limitations are related to a low efficacy at reducing transmission and the limited reproducibility, while many pointed out a paucity of data on efficacy and acceptability for these approaches.

It was possible for the responders to leave open comments. Many respondents highlighted that the question of applicability is considered to be very context-dependant, including the type of emergency and where tools are applied. Considering emergency settings in general, efficacy is considered to be the highest priority and it has also been mentioned that both IRS and LLIN are core tools recommended by the WHO while the others are not.

Regarding the better perception of LLINs over IRS, respondents highlighted LLIN’s easier implementation, however it also reported that they require a substantial investment at the community level. The supply chain—purchase and transport of commodities—takes usually more time (several months *vs* several weeks) for LLINs compared to IRS and this makes the latter easier to use in acute emergency situations. Moreover, the spectrum of insecticides used for IRS is larger than the ones used for LLIN and this can, of course, be considered as an asset to counter the threat of localised resistance patterns.

Regarding topical repellents, they are considered to rely too much of behavioural changes by the users in both emergency and non-emergency setings and there is no strong evidence of their epidemiological impact on malaria beyond personal protection.

### Combination of tools against malaria vectors

There are strong programmatic reasons for combining several tools for the control of malaria vectors in what is called Integrated Vector Management (IVM) [8, 9]. In order to determine which combinations of tools were considered to have the greatest potential in malaria control, we gathered the opinion of the participants in both contexts: emergency settings and non-emergency settings. It is important to note that our study focused on vector control measures only and did not specifically include an opportunity for a combination of vector control tools and non-vector control tools (e.g. Mass Drug Administration (MDA)).

#### In emergency settings

**Fig. 5:**
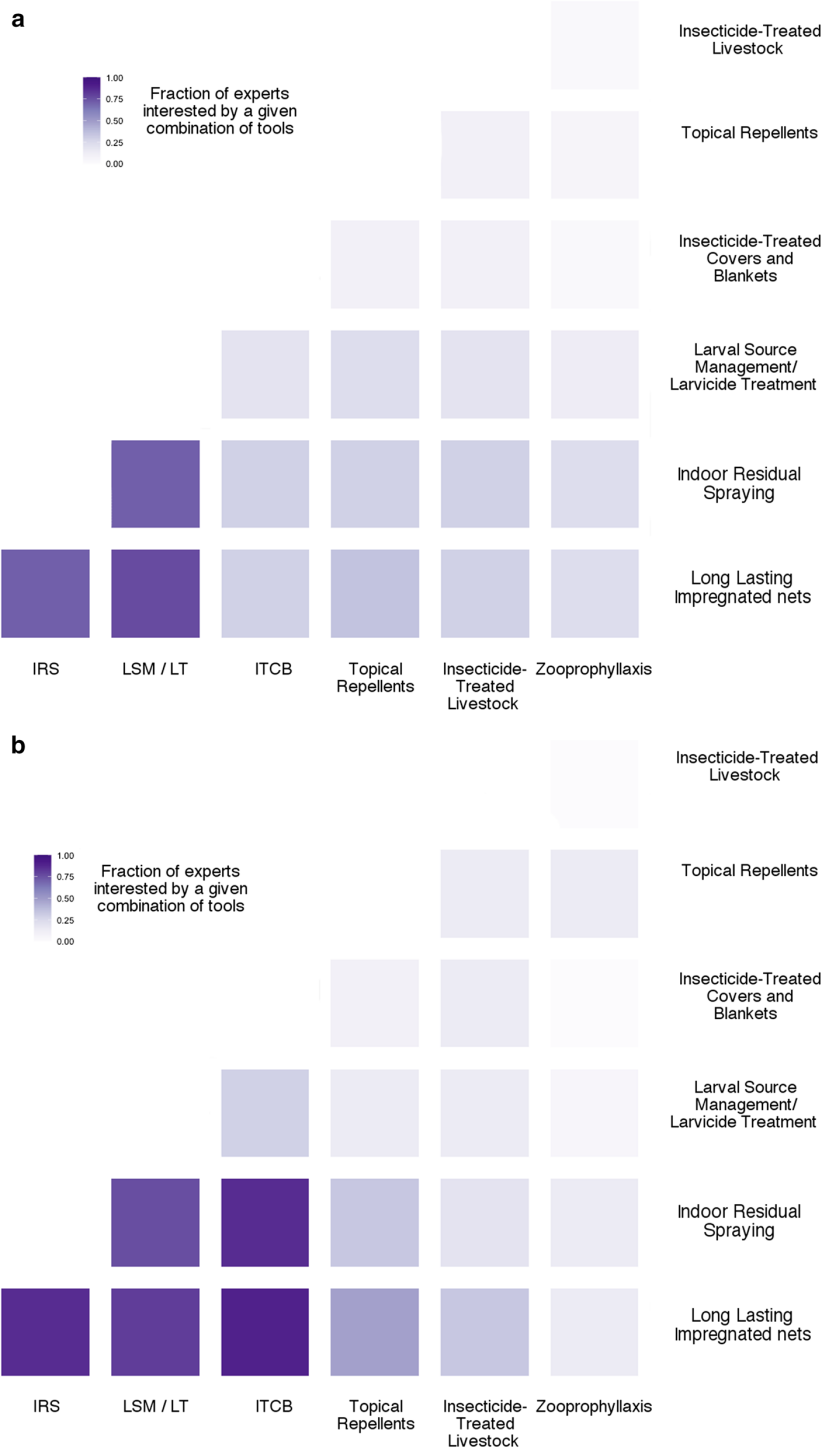
Interest of the combination of the current control tools in emergency settings. Responses are presented for all the experts (**a**) as well as for the ones (21) that have been declaring being experienced in emergency settings (**b**). *LLIN* Long-Lasting Impregnated Nets; *IRS* Indoor-Residual Spraying; *LSM*/*LT* Larval Source Management/Larvicide Treatment; *ITCB* Insecticide-Treated Covers and Blankets; *TopRep* Topical Repellents; *ITLK* Insecticide-Treated LivestocK; *Zoo* Zooprophyllaxis

**Fig. 6:**
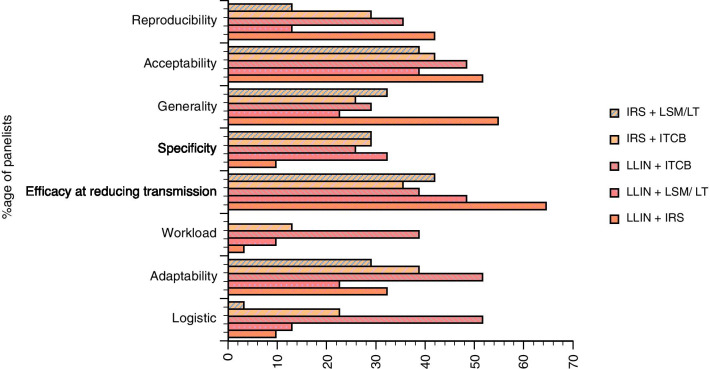
Characteristics of the different combinations of tools used in malaria vector control according to the responders

In emergency settings there is a strong interest in favour of several combinations always involving LLIN and IRS (Fig. 5a, b) and both together or in combination with LSM/LT or ITCB. This consensus on the combination is supported on the basis of a number of criteria (Fig. 6) among which acceptability and efficacy at reducing transmission are the major ones. There was a strong consensus on the need to take into account the settings specificity, the heterogeneity of the situation but also the efficiency of combinations in these settings. Regarding the implication of ITCB in combination with LLIN or IRS, this was considered as advantageous in emergency settings because of its transportability, flexibility as long as its delivery and the possibility to have a central supply are ensured.

#### In non-emergency settings

**Fig. 7:**
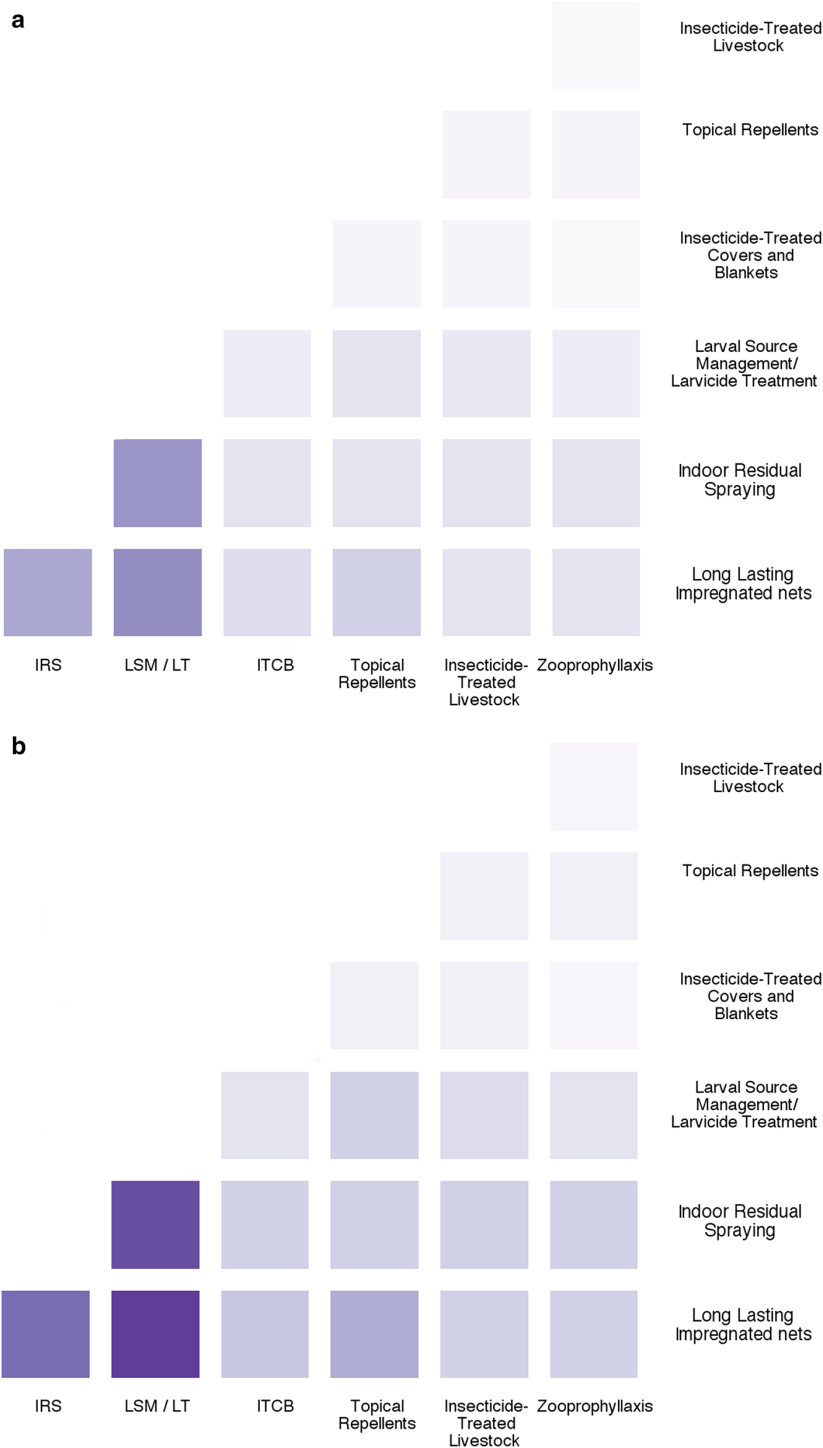
Interest of the combination of the current control tools in non-emergency settings. Responses are presented for all the experts (**a**) as well as for the ones (21) that have been declaring being experienced in emergency settings (**b**). *LLIN* Long-Lasting Impregnated Nets; *IRS* Indoor-Residual Spraying; *LSM*/*LT* Larval Source Management/Larvicide Treatment; *ITCB* Insecticide-Treated Covers and Blankets; *TopRep* Topical Repellents; *ITLK* Insecticide-Treated LivestocK; *Zoo* Zooprophyllaxis

The most valued combinations of tools in non-emergency settings involve LLIN and IRS both together or in combination with LSM/LT (Fig. 7a, b). ITCB do not appear of interest in non-emergency settings, their use and characteristics being considered more adapted to people on the move.

When asked to comment about the combination of tools, it appears that a key issue is the use of a ‘deployment package’ tailored or adapted to the situations with different human and vector behaviours. Thus LSM is considered to be a tool of interest for specific situations where surface water is limited, accessible and where breeding sites are few, fixed and findable as mentioned in WHO policy [10]. However, a major problematic aspect highlighted was the lack of robust data on the epidemiological efficacy for several of the tools mentioned (treated blankets, larval source management) compared to IRS and LLINs. Concerns were also expressed about the lack of evidence of any epidemiological impact of the combination of IRS and LLINs (or any other combinations) provided that one intervention is implemented at high coverage; an associated increase in the cost of vector control when tools are combined is also mentioned with the uncertainty of its cost-effectiveness compared to the use of one tool only if associated with behavioural changes to ensure its full efficacy.

## Research priorities in malaria vector control

**Fig. 8:**
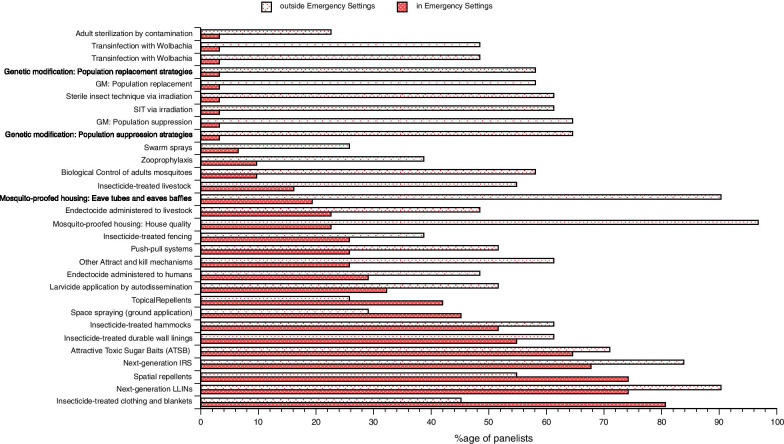
Research priorities for malaria vector control in emergency and non-emergency settings

Apart from determining which of the tools currently being used in various malaria endemic contexts were considered as most relevant by specialists in malaria control, we set out to determine opinions about research priorities in this field (Fig. 8). This reveals that in emergency settings as well as in non-emergency settings, research to demonstrate means of improving currently existing tools (next generation LLINs, next generation IRS, ATSB…) received the strongest support. When it comes to house modification, whether its structural improvement [11] or the use of device such as eave tubes [12], it appeared as a major priority (> 90%) but only in non-emergency settings.

Regarding ATSB and Spatial Repellents, most responders considered that, because they are still being investigated, these tools still needed to be tested in large emergency settings. This is related not only to their efficacy but also to the practical aspects of their delivery, the associated logistics and workload demand.

Lastly, concerning genetic approaches ranging from sterile-insect (SIT) via irradiation to genetic modification or transinfection with *Wolbachia*, there was some interest and support in non-ES (50–70%) but less than 5% of the responders consider the developmental need of such approaches for malaria vector control in emergency settings. There are a number of reasons for this, in the case of SIT, the need for mosquito mass-rearing facilities [13] is considered a serious obstacle for the deployment of such approach in emergency settings. Regarding the other approaches (GM or *Wolbachia*) they are still tools under development or at the early experimental stage for which there is a clear lack of evidence of efficacy as well as the absence of solid estimates of the speed of their impact. Concerning their eventual use, the responders mentioned a number of issues related to the question of the ease of delivery and the potential associated fear by the concerned populations but also to the difficult regulatory issues in emergency settings. There are also concerns related to the monitoring and the evaluation of the intervention and the speed at which they could have an impact in emergency settings.

**Fig. 9:**
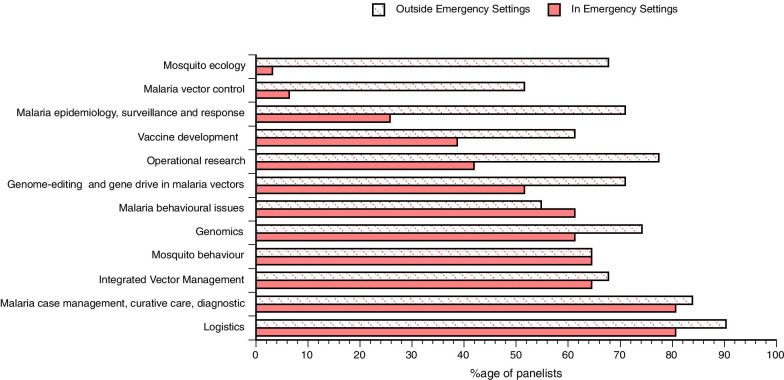
Research priorities for malaria control in emergency and non-emergency settings

Regarding research priorities in malaria control outside of vector control, responders highlighted that a breadth of research agenda topics exists and there was a strong emphasis towards practical aspects and proven tools for emergency settings (Fig. 9). Case management, diagnostics, chemoprevention and intervention logisitics were considered to be of prime importance in emergency settings compared to other research areas. Integrated vector management was ranked quite highly as a priority including in emergency settings; this was consistent with the feedback from several responders who mentioned the lack of epidemiological evidence for a number of tools or their combinations. Thus, what appears crucial is the need to know the effectiveness of tools to assemble evidence in public health from field trials in non-emergency settings before being considered in emergency settings. Interestingly, both mosquito ecology and malaria vector control were considered with some level of importance by more than two-third of our participants.

## Discussion

An expert participation rate of 46.25% (37/80) in the first round and a final participation level of 38.75% (31/80) were within acceptable boundaries for Delphi surveys, where ranges of between 10–80% are considered to be acceptable, and the participation rate was favourable [14] in comparison with other surveys on malaria research and global health [4, 5] which obtained the complete participation of 49 (48.5% of the originally contacted) and 19 experts respectively.

Interestingly we only had 6 participants dropping out between round 1 and 2, suggesting that the engaged participants were interested and committed to the topic. Regarding the repartition of our experts, subjects with all types of expertise participated with higher coverage for the technical and programmatic experts. This is consistent with the topic of malaria control in complex settings and removes the bias of a group largely working in academic malaria research. Regarding the geographic area, Africa’s representation was proportional to its high share of global malaria burden..

One of the strengths of the Delphi survey approach is that it allows us to obtain the opinion of experts in a manner that could provide a better understanding on both convergence and divergence of opinion thanks to an iterative approach where members can provide opinions and reassess them at the next stage when they are informed of the input from the other participants. Obviously, the major drawback is the lack of statistical analysis, thus limiting generalization. However, as our survey focused on malaria emergency settings that is quite a narrow topic within malaria research, we believe that we have been able to capture a fair estimate of the opinions circulating in the community of experts focusing on this particular aspect of the field and have also been avoiding the pitfall of an unevenly distributed knowledge about malaria control in specific conditions.

Regarding the results for malaria control, it appears that a common denominator in emergency and non-emergency settings is the need for efficient tools that are supported by robust scientific evidence. It is important to notice that the idea of ameliorating our current vector control echoes a comment expressed in a previous Delphi study on malaria “*our current best tools are still also our oldest*” [4].

## Conclusion

Perhaps unsurprisingly, our study highlights the need for epidemiological evidence and not only for entomological data. The lack of epidemiological evidence is a critical aspect for experts and this goes along with the need for more evidence when tools are combined to ensure an efficient integrated vector management approach. This is in accordance with the approach by the Vector Control Advisor Group (VCAG) at the WHO, which has now requested two trials with entomological and epidemiological endpoints in contrasted epidemiological settings when evaluations of novel tools for vector control are conducted.

While there appears to be little interest for innovative approaches in emergency settings, this seems to be mainly because they are still under development and lacking evidence of efficacy. This attitude appears to be compounded by social aspects (public engagement, acceptance, governance) related to novel or high-tech approaches that are challenging in both emergency and non-emergency settings. Fear can indeed be disastrous for public health and vector control programmes [15] especially in difficult contexts or outbreak situations where confidence and trust are essential for the effectiveness of the response.

## Supplementary Information

**Additional file 1**. Invitation sent to participate in the Delphi survey.

**Additional file 2**. Questionnaire used for the 1st round of the Delphi survey.

**Additional file 3**. Questionnaire used for the 2nd round of the Delphi survey.

## Data Availability

All data analyzed during this study are available from the corresponding author on reasonable request.
