# Supplementary material for: Malaria vector control tools in emergency settings: What do experts think? Results from a DELPHI survey
Source: Confl Health. 2021 Dec 20;15:93. doi: 10.1186/s13031-021-00424-y (PMC8686338; doi:10.1186/s13031-021-00424-y)
Supplement: Supplementary file 1 — Additional file 1. Invitation sent to participate in the Delphi survey. [file 13031_2021_424_MOESM1_ESM.rtf]

To : …
Title : Malaria Vector Control - Expert Opinion Survey by MSF Spain

Dear …,
 
I am contacting you on behalf of a team of members of Médecins Sans Frontières Spain (MSF OCBA: https://www.msf.es/) to invite you to participate in an expert opinion survey regarding malaria vector control. Considering the large burden of morbidity and mortality that malaria causes in the emergency contexts where MSF works, the organisation is currently exploring innovative vector control tools for Malaria.
 
The project aims at reviewing the utility of existing tools within the emergency context setting, and then developing a better understanding of emerging vector control tools (including Genetically Modified Mosquitoes) that may address malaria-related morbidity and mortality. It will also build MSF's knowledge, engage vulnerable populations and investigate ethical and intellectual property implications with regards to this dossier.
 
One of the approaches we have chosen to better understand the subject is a survey following the Delphi methodology. This methodology draws in expert opinion from approximately 30 to 50 subject experts in order to evaluate several aspects related to malaria vector control. We would like to consider 4 majors topics:
1.	the utility and sustainability of current vector control tools, both in and outside emergency settings
2.	the feasibility, utility and challenges of emerging vector control tools, both in and outside emergency settings
3.	the current and unmet research priorities in malaria vector control.
4.	the current and unmet research priorities in malaria control in general.
The survey can be completed electronically, and should take no more than 20 minutes. If you agree taking part in this project, please let me know by emailing me: christophe.boete@barcelona.msf.org and you should receive a request to fill the survey via the Questionpro platform very soon. As part of the methodology, once we receive the replies, they will be anonymised and collated. If there remains a wide opinion range on certain issues, a second survey will be sent out where the amalgamated opinions from all the experts are provided to explore whether further consensus can be reached. The surveys will be separated by a 6-week period.
 
If you are aware of other expert colleagues who may be interested in participating in this exercise, we would appreciate you sharing their contact details. We thank you in advance for your interest, your time and your support and I am looking forward to hearing back from you.
 
Best regards,
 
Christophe Boëte, Delphi survey coordinator
Silvia Moriana, Innovation and Transformation Lead, MSF OCBA
Will Robertson, Deputy Director of Operations, MSF OCBA
