# Supplementary material for: Malaria vector control tools in emergency settings: What do experts think? Results from a DELPHI survey
Source: Confl Health. 2021 Dec 20;15:93. doi: 10.1186/s13031-021-00424-y (PMC8686338; doi:10.1186/s13031-021-00424-y)
Supplement: Supplementary file 2 — Additional file 2. Questionnaire used for the 1st round of the Delphi survey. [file 13031_2021_424_MOESM2_ESM.pdf]

Survey: Delphi_MSF

Delphi_MSF

Dear colleague,

On behalf of a team of members of Médecins Sans Frontières Spain (MSF OCBA: https://www.msf.es/) I thank you for accepting to participate in an expert opinion survey regarding malaria vector control. Considering the large burden of morbidity and mortality that malaria causes in the emergency contexts where MSF works, the organisation is currently exploring innovative vector control tools for Malaria.

The project aims at reviewing the utility of existing tools within the emergency context setting, and then developing a better understanding of emerging vector control tools (including Genetically Modified Mosquitoes) that may address malaria-related morbidity and mortality. It will also build MSF's knowledge,engage vulnerable populations and investigate ethical and intellectual property implications with regards to this dossier.

One of the approaches we have chosen to better understand the subject is a survey following the Delphi methodology. This methodology draws in expert opinion in order to evaluate several aspects related to malaria vector control. We would like to consider 4 majors thematics:

i) the utility and sustainability of current vector control tools, both in and outside emergency settings
ii) the feasibility, utility and challenges of emerging vector control tools, both in and outside emergency settings
iii) the current and unmet research priorities in malaria vector control.
iv) the current and unmet research priorities in malaria control in general.

The survey should take no more than 20 minutes.

Important point:
When the issue of 'emergency' is mentioned in the survey, we use it in the following acception (as accepted by the WHO in *Environmental health in emergencies and disasters: a practical guide*, WHO, 2002). Complex emergencies are situations of disrupted livelihoods and threats to life produced by warfare, civil disturbance and large-scale movements of people, in which any emergency response has to be conducted in a difficult political and security environment. Complex emergencies combine internal conflict with large-scale displacements of people, mass famine or food shortage, and fragile or failing economic, political, and social institutions. Often, complex emergencies are also exacerbated by natural disasters.

As part of the methodology, once we receive the replies, they will be anonymised and collated. If there remains a wide opinion range on certain issues, a second survey will be sent out where the amalgamated opinions from all the experts are provided to explore whether further consensus can be reached. The surveys will be separated by a 6-week period.

If you are aware of other expert colleagues who may be interested in participating in this exercise, we would appreciate you sharing their contact details. We thank you in advance for your interest, your time and your support and I am looking forward to hearing back from you.
Please start with the survey now by clicking on the Continue button below.

---

* ***What is your major area of work? (please tick the corresponding box)***

- ○ Epidemiology, case management and control
- ○ Ethics in Health
- ○ Health Systems research and socio-economic aspects of disease research and control
- ○ Research Capacity building and strengthening
- ○ Vector control and biology
- ○ Other (please name)

---

*
Which type(s) of expertise do correspond to most of your experiences and activities: (multiple choice is possible)

- ☐ *Academic expertise*
- ☐ *Programme Expertise*
- ☐ *Technical Expertise*

If you have selected several expertises at the previous questions, please rank them *with ‘1’ being at a higher rank than ‘2’*

Academic expertise	-- Select --
Programme Expertise	-- Select --
Technical Expertise	-- Select --

* ***In which geographic area(s) are you spending most of your work time? (multiple choice is possible)***

- ☐ Africa
- ☐ Asia
- ☐ Central, South America and the Caribbean
- ☐ Europe
- ☐ North America
- ☐ Oceania

* ***Do you have experience in malaria vector control ...***

- ☐ *in **emergency** settings?*
- ☐ *in **non-emergency** settings*

* ***Do you have experience in vector control other than malaria-related ...***

☐ *in **emergency** settings?*

☐ *in **non-emergency** settings?*

Below is a list of tools currently used for the control of malaria vector. Please tick the ones that you are considering of interest in the different situations (taking into consideration its costs, its ease of implementation, its efficacy on reducing transmission …)

	**in** emergency settings	**Outside** emergency settings
1. Long Lasting Impregnated Nets (LLIN)	☐	☐
2. Indoor-Residual Spraying IRS (IRS)	☐	☐
3. Larval source management / Larvicidal treatment	☐	☐
4. Insecticide-treated clothing and blankets	☐	☐
5. Topical Repellents	☐	☐
6. Insecticide-treated livestock	☐	☐
7. Zooprophylaxis	☐	☐

If you consider the importance of combining different tools together IN Emergency Settings, please tick the box at the intersection of 2 different tools indicating which combinations are of interest according to you.

	1. LLIN	2. IRS	3. Larval source management...	4...	5...	6...	7...
1. LLIN	☐	☐	☐	☐	☐	☐	☐
2. Indoor-Residual Spraying IRS (IRS)	☐	☐	☐	☐	☐	☐	☐
3. Larval source management / Larvicidal treatment	☐	☐	☐	☐	☐	☐	☐
4. Insecticide-treated clothing and blankets	☐	☐	☐	☐	☐	☐	☐
5.Topical Repellents	☐	☐	☐	☐	☐	☐	☐
6. Insecticide-treated livestock	☐	☐	☐	☐	☐	☐	☐
7. Zooprophylaxis	☐	☐	☐	☐	☐	☐	☐

If you consider the importance of combining different tools together OUTSIDE Emergency Settings, please tick the box at the intersection of 2 different tools indicating which combinations are of interest according to you.

	1. LLIN	2. IRS	3. Larval source management...	4...	5...	6...	7...
1. LLIN	☐	☐	☐	☐	☐	☐	☐
2. Indoor-Residual Spraying IRS (IRS)	☐	☐	☐	☐	☐	☐	☐
3. Larval source management / Larvicidal treatment	☐	☐	☐	☐	☐	☐	☐
4. Insecticide-treated clothing and blankets	☐	☐	☐	☐	☐	☐	☐
5.Topical Repellents	☐	☐	☐	☐	☐	☐	☐
6. Insecticide-treated livestock	☐	☐	☐	☐	☐	☐	☐
7. Zooprophylaxis	☐	☐	☐	☐	☐	☐	☐

Below is a list of tools currently under development for vector control of malaria.

Please tick the ones that you are considered of interest in the different situations (taking into consideration costs, ease of implementation, efficacy on reducing transmission …).

	**IN** emergency settings	**OUTSIDE** emergency settings
Attractive Toxic Sugar Baits (ATSB)	☐	☐
Swarm sprays	☐	☐
Housing improvement	☐	☐
Livestock targets	☐	☐
Spatial repellents	☐	☐
Next-generation LLINs	☐	☐
Next-generation IRS	☐	☐
Sterile insect technique via irradiation	☐	☐
Topical repellents	☐	☐
Endectocide for humans	☐	☐
Transinfection with *Wolbachia*	☐	☐
Population suppression strategies via genetic modification	☐	☐
Population replacement strategies using genetically modified mosquitoes and gene drive	☐	☐

Below are different research priorities in malaria vector control.

Please tick the ones that you are considering of interest in the different situations according to its importance (taking into

consideration costs, ease of implementation, efficacy on reducing transmission …)

	**IN** emergency settings	**OUTSIDE** emergency settings
Topical Repellents	☐	☐
Spatial repellents	☐	☐
Insecticide-treated clothing and blankets	☐	☐
Insecticide-treated hammocks	☐	☐
Mosquito-proofed housing: House quality	☐	☐
Mosquito-proofed housing: Eave tubes and eaves baffles	☐	☐
Insecticide-treated livestock	☐	☐
Zooprophylaxis	☐	☐
Biological Control of adults mosquitoes	☐	☐
Transinfection with *Wolbachia*	☐	☐

	**IN** emergency settings	**OUTSIDE** emergency settings
Genetic modification: Population suppression strategies	☐	☐
Genetic modification: Population replacement strategies	☐	☐
Sterile insect technique via irradiation	☐	☐
Space spraying (ground application)	☐	☐
Attractive Toxic Sugar Baits (ATSB)	☐	☐
Endectocide administered to humans	☐	☐
Endectocide administered to livestock	☐	☐
Push-pull systems	☐	☐
Insecticide-treated durable wall linings	☐	☐
Other Attract and kill mechanisms	☐	☐

	**IN** emergency settings	**OUTSIDE** emergency settings
Adult sterilization by contamination	☐	☐
Larvicide application by autodissemination	☐	☐
Insecticide-treated fencing	☐	☐
Swarm sprays	☐	☐
Next-generation LLINs	☐	☐
Next-generation IRS	☐	☐

While research on malaria vector control is often presented as a major aspect in the future of the fight against the disease, other research thematics are currently in development. We would appreciate if you could tick the ones that you consider of importance.

	**in** emergency settings	**outside** emergency settings
Mosquito ecology	☐	☐
Mosquito behaviour	☐	☐
Genomics	☐	☐
Genome-editing and gene drive in malaria vectors	☐	☐
Malaria epidemiology, surveillance and response	☐	☐
Malaria case management, curative care, diagnostic	☐	☐
Malaria vector control	☐	☐
Malaria behavioural issues	☐	☐
Logistics	☐	☐
Operational research	☐	☐
Integrated Vector Management	☐	☐
Vaccine development	☐	☐
