# Supplementary material for: Malaria vector control tools in emergency settings: What do experts think? Results from a DELPHI survey
Source: Confl Health. 2021 Dec 20;15:93. doi: 10.1186/s13031-021-00424-y (PMC8686338; doi:10.1186/s13031-021-00424-y)
Supplement: Supplementary file 3 — Additional file 3. Questionnaire used for the 2nd round of the Delphi survey. [file 13031_2021_424_MOESM3_ESM.pdf]

Delphi_Round02

Dear colleague,

You have participated in the 1st round of the Delphi survey that I launched several months ago on behalf of a team of members of Médecins Sans Frontières Spain (MSF OCBA: https://www.msf.es/).

The project aims at reviewing the utility of existing tools for malaria control within the emergency context setting, and then developing a better understanding of emerging vector control tools (including Genetically Modified Mosquitoes) that may address malaria-related morbidity and mortality.

As you have seen in the 1st round we are considering 4 majors topics:

i) the utility and sustainability of current vector control tools, both in and outside emergency settings
ii) the feasibility, utility and challenges of emerging vector control tools, both in and outside emergency settings
iii) the current and unmet research priorities in malaria vector control.
iv) the current and unmet research priorities in malaria control in general.

The purpose of the second round is to try to gather a consensus between the remaining divergent opinions from the first round.

Just a reminder for this 2nd round :

When the issue of 'emergency' is mentioned in the survey, we assume the following definition (from the WHO in Environmental health in emergencies and disasters: a practical guide, WHO, 2002).

"Complex emergencies are situations of disrupted livelihoods and threats to life produced by warfare, civil disturbance and large-scale movements of people, in which any emergency response has to be conducted in a difficult political and security environment. Complex emergencies combine internal conflict with large-scale displacements of people, mass famine or food shortage, and fragile or failing economic, political, and social institutions. Often, complex emergencies are also exacerbated by natural disasters."

One last important point before starting the questionnaire, I am asking you to leave your name or email address in the first box below so that I can associate your answers from the 2 rounds. Once this is done, the replies will of course be anonymized and collated.

I thank you in advance for your interest, your time and your support and I am looking forward to hearing back from you.

Please start with the survey now by clicking on the Continue button below.

QuestionPro

Dear colleague,

You have participated in the 1st round of the Delphi survey that I launched several months ago on behalf of a team of members of Médecins Sans Frontières Spain (MSF OCBA: https://www.msf.es/).

The project aims at reviewing the utility of existing tools for malaria control within the emergency context setting, and then developing a better understanding of emerging vector control tools (including Genetically Modified Mosquitoes) that may address malaria-related morbidity and mortality.

As you have seen in the 1st round we are considering 4 majors topics:

i) the utility and sustainability of current vector control tools, both in and outside emergency settings
ii) the feasibility, utility and challenges of emerging vector control tools, both in and outside emergency settings
iii) the current and unmet research priorities in malaria vector control.
iv) the current and unmet research priorities in malaria control in general.

The purpose of the second round is to try to gather a consensus between the remaining divergent opinions from the first round.

Just a reminder for this 2nd round :

When the issue of ‘emergency’ is mentioned in the survey, we assume the following definition (from the WHO in Environmental health in emergencies and disasters: a practical guide, WHO, 2002).

"Complex emergencies are situations of disrupted livelihoods and threats to life produced by warfare, civil disturbance and large-scale movements of people, in which any emergency response has to be conducted in a difficult political and security environment. Complex emergencies combine internal conflict with large-scale displacements of people, mass famine or food shortage, and fragile or failing economic, political, and social institutions. Often, complex emergencies are also exacerbated by natural disasters."

One last important point before starting the questionnaire, I am asking you to leave your name or email address in the first box below so that I can associate your answers from the 2 rounds. Once this is done, the replies will of course be anonymized and collated.

I thank you in advance for your interest, your time and your support and I am looking forward to hearing back from you.

Please start with the survey now by clicking on the Continue button below.

Dear colleague,

You have participated in the 1st round of the Delphi survey that I launched several months ago on behalf of a team of members of Médecins Sans Frontières Spain (MSF OCBA: https://www.msf.es/).

The project aims at reviewing the utility of existing tools for malaria control within the emergency context setting, and then developing a better understanding of emerging vector control tools (including Genetically Modified Mosquitoes) that may address malaria-related morbidity and mortality.

As you have seen in the 1st round we are considering 4 majors topics:

i) the utility and sustainability of current vector control tools, both in and outside emergency settings
ii) the feasibility, utility and challenges of emerging vector control tools, both in and outside emergency settings
iii) the current and unmet research priorities in malaria vector control.
iv) the current and unmet research priorities in malaria control in general.

The purpose of the second round is to try to gather a consensus between the remaining divergent opinions from the first round.

Just a reminder for this 2nd round :

When the issue of ‘emergency’ is mentioned in the survey, we assume the following definition (from the WHO in Environmental health in emergencies and disasters: a practical guide, WHO, 2002).

"Complex emergencies are situations of disrupted livelihoods and threats to life produced by warfare, civil disturbance and large-scale movements of people, in which any emergency response has to be conducted in a difficult political and security environment. Complex emergencies combine internal conflict with large-scale displacements of people, mass famine or food shortage, and fragile or failing economic, political, and social institutions. Often, complex emergencies are also exacerbated by natural disasters."

One last important point before starting the questionnaire, I am asking you to leave your name or email address in the first box below so that I can associate your answers from the 2 rounds. Once this is done, the replies will of course be anonymized and collated.

I thank you in advance for your interest, your time and your support and I am looking forward to hearing back from you.

Please start with the survey now by clicking on the Continue button below.

Could you please write you name and/ or email address so that I can aggregate your answers from the 1st and 2nd round.

Regarding the interest of a variety of tools in emergency and non-emergency settings here are several major key points highlighted by the 1st round of the survey:
- There is a strong consensus about the interest of LLIN (Long-Lasting Impregnated Nets), both in emergency and non-emergency settings.
- While IRS (Indoor Residual Spraying) is considered an interesting tool in non-emergency settings for a large majority of responders, it presents a lower level of interest in emergency settings.
- There is no consensus around the use of Larval Source Management / Larvicidal Treatment (LSM/LT) in emergency settings.
- There is no consensus around the use of Topical Repellents in emergency settings.

The following questions aim at exploring these differences of opinion for each tool.

For each tool, a list of potential advantages is presented, please tick the ones you consider valid for the presented tool:

	IRS in Emergency Settings	IRS in non-Emergency Settings	LSM/LT in emergency Settings	LSM/LT in non-emergency Settings	Topical Repellents in emergency Settings	Topical Repellents in non-emergency Settings
Easy logistic implementation (including facility and community level)	☐	☐	☐	☐	☐	☐
Easy to adapt to differences in type of households targeted	☐	☐	☐	☐	☐	☐
Low associated workload	☐	☐	☐	☐	☐	☐
Efficacy in reducing transmission	☐	☐	☐	☐	☐	☐
High Specificity of the tool (only usable for specific contexts)	☐	☐	☐	☐	☐	☐
General tool in Vector control (valid in a variety of contexts)	☐	☐	☐	☐	☐	☐
Good acceptability by the population	☐	☐	☐	☐	☐	☐
Good reproducibility in large populations	☐	☐	☐	☐	☐	☐

If you have any comment about any of these tools (advantages/ disadvantages) in emergency or non-emergency settings, please let us know in the box below:

Combinations of tools are often considered with interest in malaria vector control with the idea that it could lead to a higher efficiency than the use of a single tool. Several combinations receive some support in the first round of the survey, all of them including LLIN and/ or IRS.

For the emergency settings, in the first round of the Delphi survey the following combinations are the most supported ones:

- LLIN+IRS
- LLIN+ Larval Source Management / Larvicidal Treatment (LLIN+LSM/LT)
- LLIN+Insecticide-Treated Covers and Blankets (LLIN+ITCB)
- IRS+ Insecticide-Treated Covers and Blankets (IRS+ITCB)
- IRS+Larval source management / Larvicidal treatment (IRS+LSM/LT)

For non Emergency settings, the favoured ones in the 1st round of the Delphi survey were, LLIN+LSM/LT and LLIN+IRS with more than 70% and IRS+LSM/LT with about 65%.

For the following combinations in emergency settings / non-emergency settings, please tick the listed advantage(s) that you consider valid for the given combinations:

	LLIN+IRS in Emergency Settings	LLIN+IRS in non-Emergency Settings	LLIN+LSM/LT in emergency Settings	LLIN+LSM/LT in non-emergency Settings	LLIN+ITCB in emergency Settings	IRS+ITCB in emergency Settings	IRS+LSM/LT in emergency Settings	IRS+LSM/LT in non emergency Settings
Easy logistic implementation (including facility and community level)	☐	☐	☐	☐	☐	☐	☐	☐
Easy to adapt to differences in type of households targeted	☐	☐	☐	☐	☐	☐	☐	☐
Low associated Workload	☐	☐	☐	☐	☐	☐	☐	☐
Efficacy in reducing transmission	☐	☐	☐	☐	☐	☐	☐	☐
High Specificity of the tool (only usable for specific contexts)	☐	☐	☐	☐	☐	☐	☐	☐
General tool in Vector control (valid for numerous contexts)	☐	☐	☐	☐	☐	☐	☐	☐

	LLIN+IRS in Emergency Settings	LLIN+IRS in non-Emergency Settings	LLIN+LSM/LT in emergency Settings	LLIN+LSM/LT in non-emergency Settings	LLIN+ITCB in emergency Settings	IRS+ITCB in emergency Settings	IRS+LSM/LT in emergency Settings	IRS+LSM/LT in non emergency Settings
Good Acceptability by the population	☐	☐	☐	☐	☐	☐	☐	☐
Good Reproducibility in large populations	☐	☐	☐	☐	☐	☐	☐	☐

If you have comments about any of these tools (advantages/ disadvantages) in emergency or non-emergency settings, please let us know in the box below:

While not receiving much support in non-ES, the combinations "LLIN+ITCB" and "IRS+ITCB" are considered of interest by more than half of the participants in ES.

Do you see specific advantages for these combinations in the particular contexts of Emergency Settings that they do not have in non-Emergency settings ?

Concerning the tools currently under development, next-generation LLINs and IRS are highly supported by more than 80% of the responders both in emergency and non-emergency settings. In both emergency and non-emergency settings, Attractive Toxic Sugar Baits (ATSB) and Spatial repellents receive some support with ranges from 40 and 60 %.
For ATSB and Spatial repellents, could you please tick if you consider the listed advantages valid whether in Emergency Settings and in non Emergency Settings:

	ATSB in Emergency Settings	ATSB in non-Emergency Settings	Spatial Repellents in emergency Settings	Spatial Repellents in non-emergency Settings
Easy logistic Implementation (including facility and community level)	☐	☐	☐	☐
Easy to adapt to differences in type of households targeted	☐	☐	☐	☐
Low Associated workload	☐	☐	☐	☐
Efficacy in reducing transmission	☐	☐	☐	☐
High Specificity of the tool (only usable for specific contexts)	☐	☐	☐	☐

	ATSB in Emergency Settings	ATSB in non-Emergency Settings	Spatial Repellents in emergency Settings	Spatial Repellents in non-emergency Settings
General tool in Vector control (valid for numerous contexts)	☐	☐	☐	☐
Good Acceptability by the population	☐	☐	☐	☐
Good Reproducibility in large populations	☐	☐	☐	☐

If you have any comment regarding the potential advantages/ disadvantages of ATSB or Spatial Repellents, please let us know in the box below.

Concerning novel technologies relying on high-tech and genetic approaches (SIT, transinfection with Wolbachia, Population Replacement or Population Suppression via genetic modification), they are considered of very little interest in ES (less than 10% of responders) despite some interest (between 40 and 50% of the participants) for their use in non-Emergency settings. This large discrepancy between these contexts is of strong interest for us.
What major characteristics or constraints in Emergency Settings do you see as limiting for the use and deployment of such approaches?

Among the variety of research priorities in vector control that were presented to the survey participants, there is a strong consensus on the improvement of existing tools such as LLIN, IRS and innovations related to house improvement in non-ES.
Research on ITCB and Spatial repellents receives more interest for ES than for non-ES despite the interest on research on next-generations LLIN and IRS in ES.
There is however no consensus on prioritise research in vector control for several tools that are receiving mixed interest for Emergency Settings by our responders.

Please tick the characteristics that you consider as advantages for the following research priorities on the listed tools they may improve or lead to:

	Insecticide-treated Hammock in Emergency Settings	Insecticide-treated Hammock in non Emergency Settings	Space Spraying Emergency Settings	Space Spraying in non Emergency Settings	ATSB in emergency Settings	ATSB in non emergency Settings	Treated durable wall-lining in non emergency Settings	Treated durable wall-lining in emergency Settings
Easy logistic Implementation (including facility and community level)	☐	☐	☐	☐	☐	☐	☐	☐
Easy to adapt to differences in type of households targeted	☐	☐	☐	☐	☐	☐	☐	☐
Low Associated workload	☐	☐	☐	☐	☐	☐	☐	☐
Efficacy in reducing transmission	☐	☐	☐	☐	☐	☐	☐	☐
High Specificity of the tool (only usable for specific contexts)	☐	☐	☐	☐	☐	☐	☐	☐
General tool in Vector control (valid for numerous contexts)	☐	☐	☐	☐	☐	☐	☐	☐
Good Acceptability by the population	☐	☐	☐	☐	☐	☐	☐	☐
Good Reproducibility in large populations	☐	☐	☐	☐	☐	☐	☐	☐

Please do not hesitate to give some details or complementary information in the following box about those research priorities, this would be a very valuable information for us.
